# Efficacy and safety of thread embedding acupuncture on knee osteoarthritis: A randomized, controlled, pilot clinical trial

**DOI:** 10.1097/MD.0000000000021957

**Published:** 2020-09-04

**Authors:** Purumea Jun, Chang-Hyun Han, Chang Sop Yang, Min Ji Kim, Jae Soo Kim, Cho In Lee, Jung Hee Lee, Chung A. Park, Young Joon Lee, Hyun-Jong Lee

**Affiliations:** a Clinical Medicine Division, Korea Institute of Oriental Medicine, Daejeon, Republic of Korea; b Korean Convergence Medicine, University of Science & Technology (UST), Campus of Korea Institute of Oriental Medicine, Daejeon, Republic of Korea; c Department of Acupuncture & Moxibustion, College of Korean Medicine, Daegu Haany University, Daegu, Republic of Korea; d Department of Diagnostics, College of Korean Medicine, Daegu Haany University, Daegu, Republic of Korea; e Department of Preventive Medicine, College of Korean Medicine, Daegu Haany University, Gyeongsan, Republic of Korea.

**Keywords:** efficacy, knee osteoarthritis, polydioxanone, randomized-controlled trial, thread embedding acupuncture

## Abstract

**Introduction:**

: Although there are various therapeutic methods for the treatment of knee osteoarthritis, each has its advantages and shortcomings, and a definitive treatment method is yet to be determined. This pilot study is designed to obtain basic data for a further large-scale trial as well as provide information about the feasibility of thread embedding acupuncture (TEA) with polydioxanone thread in knee osteoarthritis patients.

**Methods and analysis:**

This study is a clinical trial to evaluate the efficacy and safety of TEA for knee osteoarthritis. Forty participants will be recruited at the hospital and after randomization into 2 groups of 20 (experimental and control); they will be treated for 6 weeks. The experimental group will receive TEA treatment 6 times (1 time/week) in 6 weeks on 14 defined knee areas, and the control group, acupuncture treatments 12 times (2 times/week) in 6 weeks on 9 defined acupuncture points. The visual analogue scale (VAS) will be used for the primary efficacy assessment and Short-form McGill Pain Questionnaire (SF-MPQ), Western Ontario and McMaster Universities Osteoarthritis Index (WOMAC) will be used for the secondary efficacy assessment. The follow-ups before clinical trial, 3 weeks after procedure, 6 weeks after procedure, and 4 weeks after the end of procedure will be done to compare the degree of pain with the control group, which received the acupuncture treatment.

**Conclusion:**

The trial based on this study will provide clinical information on the efficacy and safety of TEA treatment on knee osteoarthritis.

**Trial registration number:**

KCT0004844

## 1 Introduction

Osteoarthritis is a primarily noninflammatory, degenerative joint disease characterized by progressive loss of articular cartilage, subchondral bone sclerosis, osteophyte formation, changes in the synovial membrane, and an increased volume of synovial fluid with reduced viscosity and hence changed lubrication properties.^[1]^ Knee osteoarthritis is the most common among all degenerative arthritis and the key cause of chronic disorders. The prevalence rate of knee osteoarthritis increases with age and weight.^[2]^ At around age 40, approximately 25% of the entire adult population displays motor disorders in the joints and approximately 13% and 10% of women and men over the age of more than 60 years, respectively, are afflicted with knee osteoarthritis.^[3]^

Although there are various therapeutic methods for the treatment of knee osteoarthritis in modern medicine, including life management, exercise therapy, drug treatment and surgery, and so on, each has its respective advantages and shortcomings, from the perspectives of compliance of the patients, efficacy, and side effects, and a definitive treatment method is yet to be determined.^[4]^ In particular, pharmacological treatment includes the use of aspirin, acetaminophen and nonsteroidal anti-inflammatory drugs (NSAIDs), and steroid injection into the joint. However, analgesics can induce diseases of the gastrointestinal tract such as intestinal hemorrhage, indigestion and vomiting,^[5]^ as well as hypersensitive reactions such as rash. Meanwhile, injection of steroid into the joint can help achieve temporary alleviation of the symptoms but needs to be used with utmost precautions due to the risks of destruction and infection of cartilage if injected repetitively within short periods of time.^[6]^

In Korean medicine, there have been relevant research and basic studies, including acupuncture,^[7]^ pharmacopuncture treatment,^[8]^ herbal medicine treatment,^[9]^ and moxibustion treatment,^[10]^ on knee osteoarthritis with results that demonstrate the efficacy of such treatments. Thread embedding acupuncture (TEA) among Korean medicinal therapies is an acupoint stimulation therapy being newly highlighted as a type of acupoint embedding therapy with an increase range of applications in recent years.^[11]^ Thread embedding treatment maximizes stimulation by inserting embedded threads into the acupoint, percutaneous layer, superficial muscles, meridian system, or areas that induce pain by means of needle retention action.^[12]^ In addition, it activates the meridian system, supply nutrients to muscles and peripheral nerves, and strengthening cell immunity.^[13,14]^

However, few clinical studies have been done on TEA and most of them are case studies.^[15]^ Therefore, this is a clinical study to investigate the efficacy and safety of TEA by comparing its effects with those of the ordinary acupuncture group in knee osteoarthritis, through a randomly allocated clinical trial.

## 2 Methods and analysis

### 2.1 Objective

The aims of this pilot trial are to compare pain improvement between the TEA and the acupuncture groups with knee osteoarthritis, to establish the feasibility of future TEA research, and to provide clinical evidence of the basic data before the large sample size study.

### 2.2 Study design

We will conduct a single-institution, randomized controlled pilot trial to compare the efficacy and safety of TEA with acupuncture on knee osteoarthritis.

**Figure 1:**
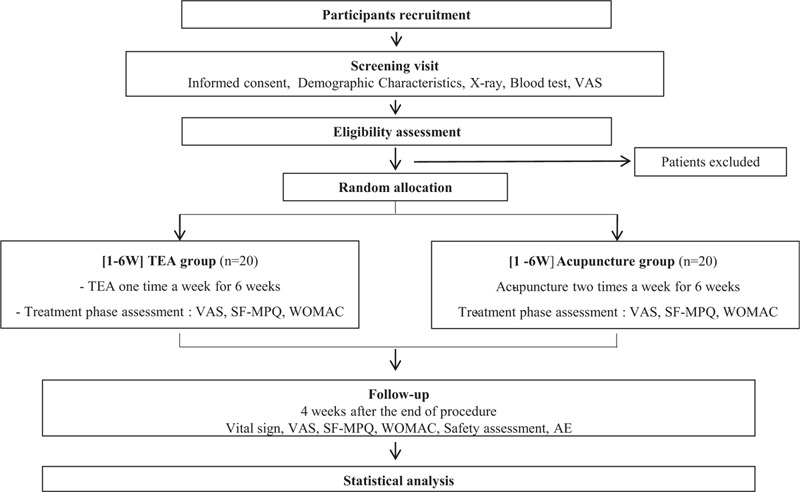
Study flow chart.

A total of 40 participants diagnosed with knee osteoarthritis will be recruited from Daegu Oriental Hospital through advertisements. The recruitment period will run from the date of Institutional Review Board (IRB) approval to December 31, 2020. The investigator will have an interview with every potential participant by telephone and schedule screening visits. During the screening visits, every potential participant will be informed in details, ample opportunities to know about all foreseeable results will be given, and written consents will be obtained from the participants. A random assignment will be performed to divide the experimental group and the control group. The probability of being assigned to the 2 groups will be the same. The experimental group will receive TEA treatment on the knee region. The control group will receive commonly used acupoints for knee osteoarthritis treatment. The visit date will be recognized 3 days before and after the visit date and carry on for 10 weeks. A flowchart of the study is illustrated in Fig. 1, and the clinical trial schedule for treatment and outcome measurement is presented in Table 1.

**Table 1:**
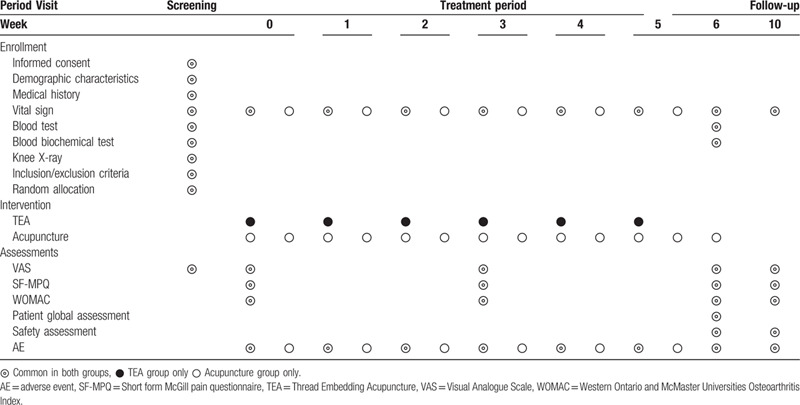
Schedule for the treatment and outcome measurements.

### 2.3 Participants

#### 2.3.1 Inclusion criteria

(1) 50 years old and above
(2) Morning stiffness to last less than 30 minutes
(3) Applicable for VAS 4-7
(4) Kellgren-Lawrence grade 2 to 3 on knee X-ray
(5) Knee pain lasting more than 3 months
(6) Consenting participants

#### 2.3.2 Exclusion criteria

(1) Those who had surgery on the knee due to osteoarthritis or other injuries
(2) Those who received intraarticular injection of phototherapy, steroids, or hyaluronic acid within the last 3 months
(3) Those with serious psychiatric problems
(4) Those in need of adrenocortical hormones or other nonsteroidal anti-inflammatory analgesic drug treatment due to other medical conditions
(5) Those with skin allergies, skin ulcers, or skin infections
(6) Insulin injecting diabetic patients
(7) Those using anticoagulants
(8) Those elevated liver function tests (ALT, AST), more than 3 times the normal value
(9) Those with abnormal findings on renal function test
(10) Those who are deemed inappropriate for the study by the person in charge

#### 2.3.3 Randomization

Block randomization will be performed, the size of the block will be 4, then the experimental and control groups will be 2:2 for each block, the randomization codes will be assigned between the groups, and will be proceeded as a pilot study. Random numbers were generated by an independent statistician. Sealed opaque assignment envelopes were used for allocation concealment. Once the clinical trial starts, each participant, who meets the selection criteria, will be assigned to the experimental and control groups according the randomization code.

### 2.4 Interventions

The participants will be randomly divided into 2 groups: the experimental group and the control group. The experimental group will receive thread embedding treatment 6 times (1 time/week) in 6 weeks on 14 defined knee areas, and the control group will receive acupuncture treatments 12 times (2 times/week) in 6 weeks on 9 defined acupuncture points.

#### 2.4.1 Experimental group

**Figure 2:**
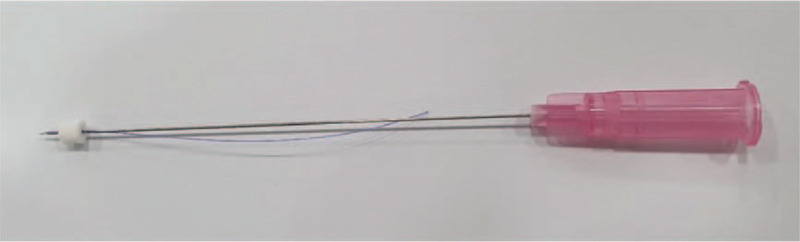
TEA used in clinical trial.

**Figure 3:**
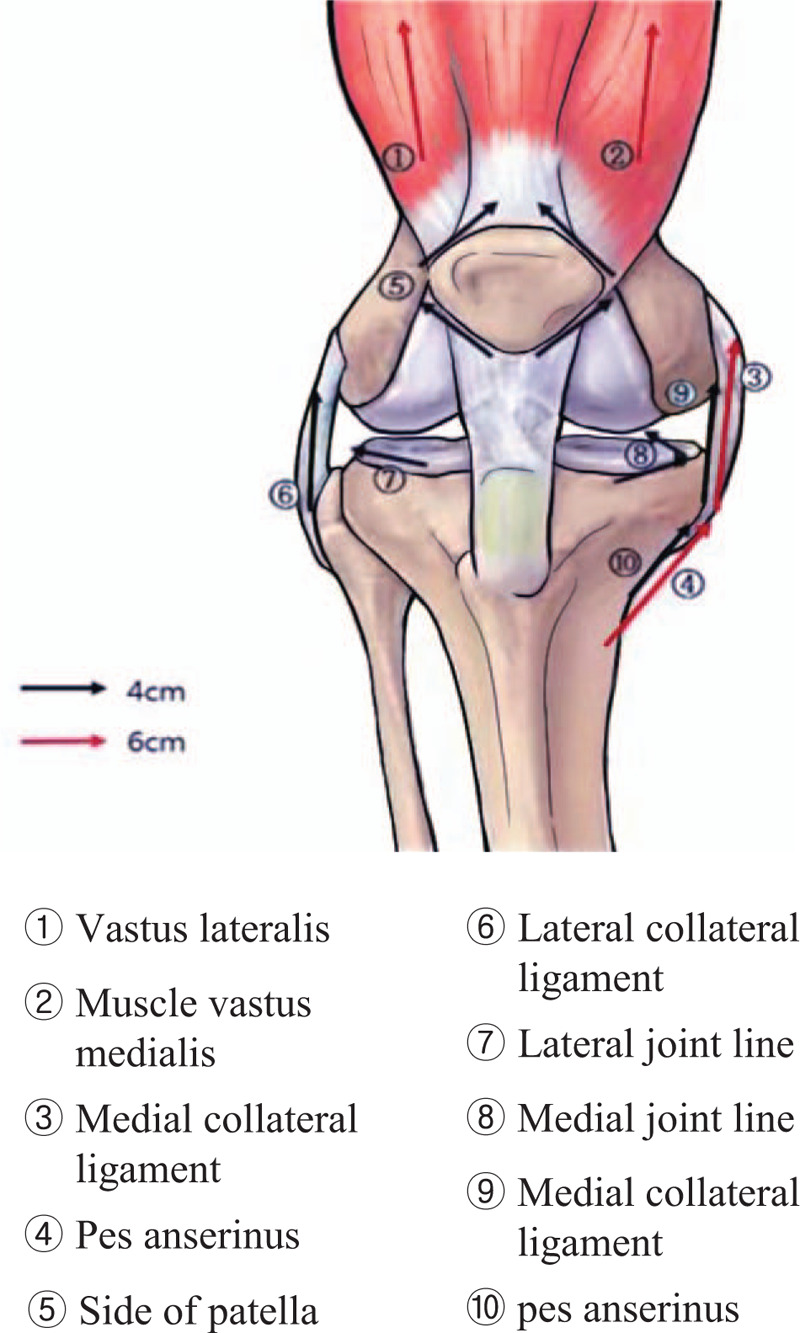
The point of TEA.

The TEA (29 gauge∗40 mm, 27 gauge∗60 mm, polydioxanone suture) was manufactured by Mircacu, feeltech Inc. Gunsan, Republic of Korea (Fig. 2). After disinfecting one side of the more painful area, KMD will perform the above embedding thread on the treatment region (Fig. 3). A total of 14 embedding threads will be used per participant. Therefore, the points of the TEA in this study will be as follows.

(1) 6 cm
  (a) Vastus lateralis. 1 horizontal needling upwards from ST34
  (b) Muscle vastus medialis. 1 horizontal needling upwards from SP10
  (c) Medial collateral ligament. 1 horizontal needling towards the direction of medial epicondyle of femur
  (d) Pes anserinus. 1 horizontal needling from Spleen 9 along the pes anserinus
(2) 4 cm
  (a) Side of patella. 4 horizontal needlings along the upper outer, upper inner, lower outer, and lower inner of patella
  (b) Lateral collateral ligament. 1 horizontal needling in the direction of lateral epicondyle of femur along the lateral collateral ligament
  (c) Lateral joint line. 1 horizontal needling toward the lateral back along the lateral joint line
  (d) Medial joint line. 2 horizontal needlings toward the medial back along the medial joint line
  (e) Medial collateral ligament. 1 horizontal needling toward the direction of medial epicondyle of femur
  (f) Pes anserinus. 1 horizontal needling from Spleen 9 along the pes anserinus

#### 2.4.2 Control group

The participants of control group will receive acupuncture intervention with 0.30 × 40 mm disposable sterilized stainless steel acupuncture needles (DongBang Acupuncture Inc., Republic of Korea), done by KMD. On one side of the more painful area, the acupuncture needles will be inserted perpendicular to a depth of 5 to 20 mm into the muscles for 20 ± 5 minutes.

In this study, the 9 acupuncture points will be used: unilateral ST34, SP09, SP10, Ex-LE04, Ex-LE02, LR08, ST36, GB33, and Ex-LE05, which will be affected as a result of acupuncture treatment on patients with knee osteoarthritis in clinical treatment.

### 2.5 Outcome measurement

A series of measurements to assess VAS for pain and short form McGill pain question (SF-MPQ), Western Ontario and McMaster Universities Osteoarthritis Index (WOMAC) will be collected before clinical trial, 3 weeks after procedure, 6 weeks after procedure, and 4 weeks after the end of procedure. Outcome measurements and the following treatment schedules are summarized in Table 1.

#### 2.5.1 Primary outcome measure

##### 2.5.1.1 Visual analogue scale score (VAS)

VAS is a 10 cm measurement instrument to determine the severity of pain.^[16]^ A number ranging from 0 to 10 on both ends of the straight line is written down in words from no pain (0) to severe pain (10). The participant will mark on a straight line according to the degree of pain, and the investigator will measure the length to evaluate the pain.

#### 2.5.2 Secondary outcome measures

##### 2.5.2.1 Short form McGill pain questionnaire (SF-MPQ)

SF-MPQ is an instrument that summarizes the popular McGill pain questionnaire with mixed VAS, descriptive scale, and present pain intensity (PPI). The descriptive scale is based on 15 selected words, including 11 items in the sensory domain and 4 items in the emotional domain. The score is set from 0 to 3 (0-no symptoms, 1-mild, 2-moderate, 3-severe). The total score is obtained by summing the individual scores of all 15 items, with higher scores indicating more severe knee pain. PPI measures current pain severity in 6 levels (0-no pain, 1-mild, 2-discomforting, 3-depressing, 4-horrible, 5-excruciating).^[17]^

##### 2.5.2.2 Western Ontario and McMaster universities osteoarthritis index (WOMAC)

The WOMAC score is a standardly used questionnaires to evaluate the pain and condition of patients with osteoarthritis of the knee and hip, including stiffness, pain, and physical functioning of the joints. It consists of 24 questions divided into 3 subscales. The index measures 2 questions for stiffness (score range 0–8), 5 questions for pain (score range 0–20), and 17 questions for physical function (score range 0–68). The total score is 96; higher scores indicate worse stiffness, pain, and functional limitations.^[18]^

### 2.6 Adverse events and safety

Adverse events are defined as undesirable and unintended signs, symptoms, and diseases that develop after treatments in this clinical study process and are not necessarily caused by this clinical study. If a serious adverse event (SAE) occurs during the study, the treatment of the participant is temporarily suspended in the clinical trial, and if continuous treatment is deemed to be dangerous to the participant and if the adverse event is related to the intervention, the participant's participation in the clinical trial is discontinued. We will confirm the safety of TEA treatment by measuring blood tests and biochemical tests at the screening and after the termination of acupuncture (visit 7, visit 13) for patients. We will check the red blood cell (RBC) count, total white blood cell (WBC), hemoglobin, hematocrit, differential count, erythrocyte sedimentation rate (ESR), platelet, aspartate aminotransferase (AST), alanine aminotransferase (ALT), blood urea nitrogen (BUN), creatinine level, C-reactive protein (CRP), prothrombin time (PT), partial thromboplastin time (PTT), serum sodium level, serum potassium level, and serum chloride level.

### 2.7 Withdrawal and dropout

All participants will have the right to withdraw from the study at any time. Participation will be ended at any phase if the subject refuses to continue, withdraws consent, or violates the inclusion or exclusion criteria. In addition, the investigator records the completion of the study by the entire participant in the clinical trial and records the reasons for the discontinuation of the procedure.

### 2.8 Recruitment

Participant recruitment announcement will be posted on website of Daegu Oriental Hospital of Daegu Haany University, inside the Daegu Oriental Hospital of Daegu Haany University, and in the subway. Before the study begins, all matters relating to the study will be explained in details, ample opportunities to know about all foreseeable results will be given, and written consents will be obtained from the participants, who meet both the inclusion criteria and the exclusion criteria, and their guardians.

### 2.9 Blinding

This is an open-label study comparing TEA with acupuncture treatment. It was not possible to design a double-blind for both the practitioner and the participant in this study, without revealing either the TEA or the acupuncture treatment, because of the intervention characteristics and area of application of the thread embedding treatment. In addition, the 2 groups have different number of visits.

### 2.10 Statistical methods

#### 2.10.1 Sample size

This clinical trial is a pilot study, and will prepare for the primary confirmation stage and basic data about prudence in the study and clinical results before the large sample size study. Hence, rather than following the separate sample counting criteria, 20 participants were selected (20% dropout rate considered) for the experimental and control groups.

#### 2.10.2 Statistical analysis

This statistical analysis is based on “Clinical Trial Statistics Guide (KFDA, 2000)” and the statistical package used is IBM SPSS Win ver. 19.0. The last observation carried forward method is used for missing data from drop-outs. For statistical significance, the significance level is set at 5%, and ITT (intention-to-treat: intent-to-treat analysis) analysis and PP (per protocol) analysis are conducted for all data analyses. ITT analysis is applied in principle.

#### 2.10.3 Analysis of baseline characteristics

##### 2.10.3.1 Analysis of full data

A descriptive analysis will be performed for the demographic characteristics (e.g., gender, age) and clinical characteristics of participants in baseline data. Quantitative data will be summarized using the mean with standard deviation (SD), and qualitative data will be summarized using the frequency and percentage.

##### 2.10.3.2 Homogeneity test of the 2 groups (experimental and control)

Homogeneity test between groups will be performed using 2-sample *t* test or Mann–Whitney *U* test for quantitative data according to the normality, and Chi-square test will be performed for qualitative data.

#### 2.10.4 Analysis of efficacy

For the primary (VAS) and secondary (SF-MPQ, WOMAC) outcomes, we will evaluate the difference of time and groups, and an interaction between the time and the groups using repeated-measure 2-factor analysis. If a significant interaction between the time and the groups was observed, we will use contrast analysis to compare individual differences between levels of the time and the groups.

For the other variables, we will evaluate the difference of the time using repeated-measure ANOVA test. When the variable measurement was implemented before and after treatment, we will calculate the change within the time, and evaluate the difference between the groups using Student *t* test as a parametric test, or Mann–Whitney test as a nonparametric test.

#### 2.10.5 Analysis of safety

Safety analysis will be performed on the participants who plan to receive TEA treatment or acupuncture treatment at least once. Frequency of adverse reaction between groups, which is related with TEA treatment and acupuncture treatment, will be assessed using Chi-square test, and proportion of participants between groups, who have experienced the adverse reaction at least 1 time, will be evaluated using Chi-square test.

### 2.11 Ethics and quality control

This study protocol has been designed according to Korean Good Clinical Practices (KGCPs) and based on the Declaration of Helsinki. This study was approved by the Institutional Review Board of Daegu Oriental Hospital of Daegu Haany University, Daegu, Republic of Korea (DHUMC-D-20001-AMD-01) and registered on the Clinical Research Information Service (identifier: KCT0004844) at Korea National Institute of Health, which is recognized as a registry in the World Health Organization's (WHO's) Network. All participants will be fully briefed on the study protocol and will be asked to provide written informed consent. Participants may withdraw from the trial at any time even after having provided consent, and their all information regarding the participants and study process is kept confidential. After the completion of this trial, an independent investigator will analyze the data for statistical analysis. All researchers will receive special training on recruitment, screening, randomization, assessment, and instructions for completing the case report form (CRF). The study findings will be published in the peer-reviewed journal.

## 3 Discussion

Osteoarthritis is a sort of joint disease characterized by joint pain and joint cartilage loss. Recently, there are various therapeutic methods for the treatment of knee osteoarthritis, each has its respective advantages and shortcomings, and a definitive treatment method is yet to be determined. Complementary alternative treatments such as acupuncture are widely used, and TEA is drawing attention among them.^[19]^ TEA has been widely used in the treatment of musculoskeletal pain by inserting polydioxanone sutures into specific regions, and by applying the physical stimulation effects and chemical stimulation effects of the TEA on knee osteoarthritis, such as muscle or subcutaneous tissue located at acupuncture points.^[13]^

Although results of several clinical studies on the effects of TEA on knee osteoarthritis were presented, the majority were case studies with no attempts to objectively evaluate the treatment effects and safety in comparison to studies on acupuncture. There have been a few randomized controlled clinical trial (RCT) researches on TEA published recently in China. However, good quality studies are hard to find. Referring to Chinese researches, the quality assessment of RCT was unclear, although the cures of the TEA for knee osteoarthritis were possible.^[20–24]^ Therefore, this pilot study is designed for the research of high-quality random trials and made considerations on the results in order to investigate the efficacy and safety of TEA.

We anticipate this pilot study to provide clinical evidence for a future large-scale and multi-institutional trial as well as information about the feasibility of such a trial.

## Author contributions

PJ and CHH designed the study and drafted the manuscript. MJK, JSK, CIL, JHL, and CAP participated in the design of the study. HJL and YJL developed the plan for the statistical analysis. CHH and CSY provided technical advice and made critical revisions to the study plan and manuscript. HJL participated in the design of the study as principal investigators and were responsible for the final decision to submit this manuscript for publication. All authors read and approved the final manuscript.
